# HIV and Low Vision: A Narrative Review

**DOI:** 10.22336/rjo.2026.04

**Published:** 2026

**Authors:** Ragni Kumari

**Affiliations:** 1 Department of Optometry, UPUMS, Saifai, Etawah, U.P., India

**Keywords:** HIV, AIDS, low vision, visual acuity, HAART, vision impairment, HIV = Human Immunodeficiency Virus, AIDS = Acquired Immunodeficiency Syndrome, HAART = Highly Active Antiretroviral Therapy, VA = Visual Acuity, VI = Vision Impairment, LV = Low Vision

## Abstract

Human Immunodeficiency Virus (HIV) continues to be a significant global public health concern, affecting over 38 million individuals globally. Although the introduction of Highly Active Antiretroviral Therapy (HAART) has led to a significant decline in HIV-related mortality and opportunistic infections, the ocular manifestations of HIV remain a major contributor to morbidity, particularly in low-resource settings. Among the spectrum of visual complications, low vision is an under-recognized yet profoundly disabling consequence that adversely impacts quality of life, independence, and mental health.

HIV-related low vision can result from multiple pathophysiological mechanisms, including direct viral damage, opportunistic infections such as cytomegalovirus (CMV) retinitis, neuro-ophthalmic complications, and HIV-associated malignancies. Clinical presentations may vary from asymptomatic retinal microvasculopathy to irreversible blindness secondary to necrotizing retinitis or optic neuropathy. The burden of ocular disease remains disproportionately high in low- and middle-income countries due to delayed diagnosis, limited access to ophthalmic care, and late initiation of antiretroviral therapy.

This narrative review provides an in-depth exploration of the epidemiology, clinical spectrum, diagnostic approaches, management strategies, and rehabilitation options for low vision in the context of HIV. Special emphasis is placed on the psychosocial challenges associated with dual stigmatization from HIV and visual impairment, as well as the pressing need for integrated models of care. Emerging innovations such as portable fundus imaging, artificial intelligence-assisted diagnostics, and sustained-release ocular drug delivery systems are also discussed as part of the evolving landscape of HIV ophthalmology.

Ultimately, a multidisciplinary approach—combining infectious disease management, ophthalmologic intervention, and low vision rehabilitation—is essential to address the complex needs of HIV-positive individuals at risk for or living with low vision. Early recognition and holistic care can mitigate disability, enhance functional outcomes, and improve overall quality of life in this vulnerable population.

## Introduction

Human Immunodeficiency Virus (HIV) infection remains one of the most significant public health challenges of the 21st century. Despite remarkable progress in antiretroviral therapy and global health initiatives, the burden of HIV continues to be substantial, particularly in low- and middle-income countries. While HIV is primarily known for its immunosuppressive effects and its progression to Acquired Immunodeficiency Syndrome (AIDS), its impact extends far beyond systemic infections. The ocular complications associated with HIV are often overlooked, yet they can lead to serious visual impairment and permanent disability if not promptly diagnosed and managed [1].

### Epidemiology of HIV

Globally, more than 38 million individuals are living with HIV, with Sub-Saharan Africa accounting for approximately two-thirds of all cases. According to UNAIDS, in 2023 alone, there were an estimated 1.3 million new infections and around 630,000 AIDS-related deaths. Significant disparities persist in terms of access to prevention, diagnosis, and treatment. The introduction of Highly Active Antiretroviral Therapy (HAART) in the mid-1990s dramatically improved life expectancy and quality of life for people living with HIV (PLWH). However, the extended survival of PLWH has led to an increased recognition of chronic complications, including those affecting the visual system.

Before HAART became widely available, ocular complications were reported in up to 70% of patients with AIDS, with cytomegalovirus (CMV) retinitis being the most common cause of blindness. Although the incidence of such infections has declined significantly in well-resourced settings, vision-threatening ocular disease remains a concern in patients with delayed diagnosis, poor adherence to therapy, or inadequate immune recovery. In under-resourced health systems, where ART coverage is inconsistent, the prevalence of low vision secondary to HIV remains unacceptably high [1].

### Importance of Visual Health in HIV

Vision is a crucial component of overall health, influencing communication, mobility, education, and employment. In people living with HIV, visual health has a multidimensional impact. First, ocular manifestations may serve as early markers of immunosuppression or opportunistic infections, offering diagnostic clues that could lead to timely therapeutic interventions. Second, visual impairment in HIV-infected individuals is associated with a disproportionate reduction in quality of life due to its additive burden on physical, emotional, and socioeconomic well-being [1,2].

Low vision, defined as visual impairment not correctable by conventional means such as glasses or surgery, can result from a spectrum of HIV-related ocular diseases, including HIV retinopathy, CMV retinitis, neuro-ophthalmic complications, and opportunistic infections like toxoplasmosis or herpetic retinitis. In children, HIV-associated ocular disease can also disrupt visual development and academic performance. Additionally, dual stigmatization—arising from both HIV status and visual disability—can severely restrict social participation and mental health resilience.

Despite these implications, visual health is not routinely prioritized in HIV care protocols, especially in settings where health systems are already overburdened. The lack of integrated ophthalmologic services within HIV clinics leads to delays in diagnosis, missed opportunities for prevention of blindness, and a failure to address the rehabilitative needs of those already affected.

This review highlights the urgent need for early recognition, comprehensive management, and structured vision rehabilitation in the context of HIV. By focusing on the intersection of HIV and low vision, we aim to bring attention to a neglected but essential aspect of holistic HIV care.

### Ocular Manifestations of HIV

HIV infection affects nearly every organ system, including the visual system. Ocular complications may present at any stage of HIV infection, often reflecting the severity of immunosuppression. The eye may serve as an early site of HIV-related disease, and in some cases, ocular signs may precede systemic symptoms. Ocular manifestations can be broadly categorized into three groups: direct effects of HIV, opportunistic infections, and neuro-ophthalmic or malignancy-related complications. These complications may lead to temporary or permanent visual impairment and are particularly consequential in settings with limited ophthalmic care [2].

#### Direct Effects of HIV

The direct impact of HIV on ocular tissues is multifactorial, involving both the virus itself and immune dysregulation. The most common direct manifestation is HIV retinopathy, observed in up to 50-70% of patients with advanced immunosuppression. This condition is characterized by cotton wool spots, retinal hemorrhages, and microaneurysms, reflecting a microvasculopathy similar to that observed in diabetes or hypertension. Although often asymptomatic, extensive retinopathy may cause visual disturbances if it involves the macula.

Other direct ocular effects include [3-5]:

- Conjunctival microvasculopathy - a benign but common finding, visible as vascular dilation and tortuosity.
- Keratoconjunctivitis sicca (dry eye syndrome) - due to HIV-induced lacrimal gland dysfunction.
- Uveitis - occasionally immune-mediated, especially during immune recovery uveitis (IRU) following HAART initiation.

While these direct effects are generally less vision-threatening compared to opportunistic infections, they may serve as indicators of systemic HIV progression.

#### Opportunistic Infections

The immunosuppressed state of advanced HIV/AIDS creates susceptibility to a wide array of opportunistic ocular infections, many of which can result in irreversible vision loss if not promptly treated.

1. Cytomegalovirus (CMV) Retinitis: The most common opportunistic ocular infection in HIV, CMV retinitis typically occurs in individuals with CD4 counts < 50 cells/mm^3^. It presents as necrotizing retinitis with retinal whitening, hemorrhages, and vascular sheathing—often described as a “pizza” or “cheese and ketchup” fundus appearance. Without treatment, CMV can cause retinal detachment and permanent blindness.
2. Toxoplasma Chorioretinitis: Caused by reactivation of latent *Toxoplasma gondii*, this infection may present with focal necrotizing retinochoroiditis, vitritis, and severe pain. Lesions often occur adjacent to old scars, and visual prognosis varies by site of involvement.
3. Herpes Zoster Ophthalmicus (HZO): Resulting from varicella-zoster virus reactivation, HZO can involve the cornea, conjunctiva, uvea, and retina. The hallmark is a painful vesicular rash in the trigeminal dermatome with ocular involvement in up to 50% of cases. Complications such as neurotrophic keratitis or secondary glaucoma may contribute to vision loss.
4. Fungal and Bacterial Infections: Candida, Aspergillus, and Mycobacterium tuberculosis can cause endophthalmitis, choroiditis, or retinal abscesses. These infections are more frequent in late-stage AIDS and are often associated with poor visual prognosis.
5. Syphilitic Uveitis: *Treponema pallidum* infection is seeing a resurgence among PLWH. Ocular syphilis can cause panuveitis, retinitis, and optic neuritis. It is treatable with systemic penicillin but requires early recognition.

These infections not only impair vision directly but may also reflect life-threatening systemic dissemination, underscoring the diagnostic and prognostic importance of ophthalmic findings in HIV.

#### Neuro-Ophthalmic and Malignancy-Related Complications

Neuro-ophthalmic disorders are increasingly recognized in HIV, particularly in patients with central nervous system involvement. These complications often arise from intracranial infections, malignancies, or immune-mediated injury.

1. Optic Neuropathy: Optic nerve dysfunction may be due to CMV, cryptococcus, tuberculosis, or HIV itself. Visual acuity loss, visual field defects, and color desaturation are common features. In some cases, optic neuritis may occur as part of immune reconstitution inflammatory syndrome (IRIS) after initiation of HAART.
2. Cranial Nerve Palsies: Involvement of cranial nerves III, IV, and VI may result from meningitis or intracranial mass lesions such as toxoplasmosis or lymphoma. Patients typically present with diplopia, ptosis, and gaze palsies.
3. Visual Cortex Involvement: Progressive multifocal leukoencephalopathy (PML), a demyelinating condition caused by the JC virus, may lead to cortical blindness, especially in late-stage HIV.
4. HIV-Associated Malignancies: Several neoplasms associated with HIV can affect the eye:

- Kaposi Sarcoma - involving the eyelids or conjunctiva.
- Non-Hodgkin Lymphoma - can present with orbital or intraocular mass lesions.
- Intraocular Lymphoma - masquerading as chronic uveitis and leading to significant visual decline.

These neuro-ophthalmic and oncologic conditions may present subtly but carry grave prognoses without multidisciplinary management.

Together, these ocular complications underscore the necessity for routine ophthalmic evaluation in people living with HIV. Timely recognition and treatment can prevent irreversible visual loss and may even reveal systemic conditions warranting urgent intervention.

### Epidemiology and Burden of HIV-Related Low Vision

Low vision, defined as a permanent visual impairment that is not fully correctable by spectacles, contact lenses, medication, or surgery, affects an estimated 246 million people globally. In people living with HIV (PLWH), visual disability is an underrecognized but growing concern. With improved access to antiretroviral therapy (ART) and longer life expectancy, chronic ocular complications—many of which are irreversible—have emerged as significant contributors to the global burden of low vision [4-6].

#### Global Burden of HIV-Related Vision Impairment

Although precise data on the prevalence of HIV-associated low vision is limited, existing studies indicate that up to 70% of patients with AIDS may develop ocular manifestations at some point in their disease course, particularly in the absence of antiretroviral therapy. In regions with high HIV prevalence and poor access to eye care, such as sub-Saharan Africa, Southeast Asia, and parts of Latin America, vision loss due to HIV-related retinopathy, CMV retinitis, or optic neuropathy is not uncommon.

In the pre-HAART era, CMV retinitis alone accounted for 20-40% of blindness among AIDS patients. While this figure has significantly declined in high-income countries due to effective viral suppression, it remains alarmingly high in resource-limited settings where routine ophthalmologic screening and antiviral therapy may be unavailable or unaffordable. A study in Zambia reported visual impairment in 30% of HIV-positive adults, with ocular infections, optic neuropathy, and HIV retinopathy as leading causes.

#### Low Vision in Children Living with HIV

Pediatric HIV poses unique challenges in ocular care. Children infected perinatally are at risk of visual developmental delays due to cortical visual impairment, nystagmus, refractive errors, or optic nerve hypoplasia. Opportunistic infections such as CMV, toxoplasmosis, or tuberculous meningitis may affect the retina and optic nerve early in life. Since vision is critical to learning and cognitive development, the consequences of undiagnosed or uncorrected visual impairment can be profound in this population [7-9].

In low-resource countries, where neonatal and early childhood screening is limited, vision loss in HIV-positive children often goes undetected until it manifests as academic underperformance or behavioral issues. Studies from India and South Africa have documented reduced visual acuity and contrast sensitivity in HIV-infected children, highlighting the need for early ophthalmic assessment as part of routine pediatric HIV care.

#### Impact on Quality of Life and Function

Low vision in PLWH significantly compromises independence, social participation, and occupational performance. Unlike vision impairment from age-related causes (e.g., cataract or macular degeneration), HIV-associated low vision tends to affect younger, economically active individuals, further amplifying the socioeconomic burden. The co-existence of HIV and visual disability often results in dual stigmatization, leading to social isolation, loss of employment, and deteriorating mental health.

Furthermore, access to vision rehabilitation services is frequently limited in low-income countries. Many patients with HIV-related low vision remain undiagnosed or untreated due to a lack of awareness among healthcare providers, a shortage of trained eye care professionals, and competing health priorities in HIV programs.

#### Barriers to Care

Multiple structural, systemic, and individual barriers hinder effective management of low vision in PLWH:

- Lack of routine ophthalmologic screening in HIV clinics, even in high-risk patients with low CD4 counts.
- Delayed presentation due to poor symptom recognition or fear of stigma.
- Limited availability of low vision aids, rehabilitation centers, or support systems.
- Fragmented care pathways, where eye care is disconnected from HIV services.

The result is a substantial unmet need for prevention, detection, and management of visual impairment in this population. Integrating vision care into HIV management frameworks is essential to improve outcomes and reduce disability.

#### Disparities by Geography and Gender

Epidemiological data suggest that women, particularly in sub-Saharan Africa, may experience a disproportionately higher burden of both HIV and untreated visual impairment. Gender-based inequalities in healthcare access, economic dependence, and cultural norms may limit women’s ability to seek or afford eye care services [10].

Geographically, the contrast between low- and high-income countries is stark. In wealthier regions, ocular complications are promptly managed with antiviral therapies and surgical interventions, whereas in poorer settings, patients often present with advanced disease and limited treatment options.

### Detailed Clinical Spectrum Leading to Low Vision in HIV

Low vision in people living with HIV (PLWH) is typically the consequence of a range of ocular complications affecting the retina, optic nerve, and ocular adnexa. These conditions may arise from direct HIV infection, opportunistic pathogens, immune-mediated inflammation, or HIV-associated malignancies. Each of these conditions carries distinct pathophysiology, clinical presentations, and prognostic implications. This section provides an in-depth overview of the major ocular entities that may lead to permanent visual impairment or blindness in HIV-positive individuals [11,12].

1. HIV Retinopathy: HIV retinopathy is the most common ocular manifestation of HIV and can occur in up to 70% of individuals with advanced disease. It is primarily a non-infectious microangiopathy caused by direct effects of HIV on the retinal microvasculature, compounded by immune complex deposition and capillary non-perfusion. Clinical features include cotton wool spots (nerve fiber layer infarcts), intraretinal hemorrhages, microaneurysms, vascular sheathing, and tortuosity. These findings are typically bilateral and often asymptomatic, but visual acuity can be affected when lesions involve the macula or when concurrent opportunistic infections occur. Though HIV retinopathy itself is usually not sight-threatening, it can be a harbinger of severe immunosuppression and signal the need for closer systemic and ophthalmic evaluation.
2. Cytomegalovirus (CMV) Retinitis: CMV retinitis remains the leading cause of blindness in AIDS patients, especially in individuals with CD4 counts below 50 cells/mm^3^. The virus reactivates from latency under conditions of profound immunosuppression and causes necrotizing retinitis. Clinical features may include painless, progressive visual loss, floaters, scotomata, or peripheral visual field defects. Fundoscopic features: full-thickness retinal necrosis, hemorrhages, “frosted branch” perivascular sheathing, and retinal detachment. Without prompt antiviral therapy (e.g., ganciclovir, valganciclovir, foscarnet), the infection can progress rapidly, leading to bilateral blindness within weeks. With the introduction of HAART, incidence rates have declined significantly in high-income countries; however, CMV retinitis remains prevalent in resource-poor settings.
3. Ocular Toxoplasmosis: *Toxoplasma gondii* is an intracellular protozoan that causes chorioretinitis, particularly in immunocompromised individuals. Reactivation of latent infection is common in AIDS patients, and lesions are often bilateral, extensive, and recurrent. Clinical features include focal or multifocal necrotizing retinochoroiditis, vitritis (“headlight in the fog” appearance), retinal vasculitis and perivasculitis, and associated optic neuritis or retinal detachment. Lesions involving the macula or optic disc carry a high risk of permanent visual impairment. Treatment usually consists of pyrimethamine, sulfadiazine, and folinic acid, often combined with corticosteroids to reduce inflammation.
4. Herpes Zoster Ophthalmicus (HZO): HZO arises from reactivation of varicella-zoster virus (VZV) in the ophthalmic branch of the trigeminal nerve. It tends to be more severe in HIV-positive individuals and can involve multiple ocular structures. Ocular involvements are keratitis (epithelial and stromal), uveitis (anterior or panuveitis), secondary glaucoma, optic neuritis, or retinal necrosis. The hallmark is a painful, vesicular rash along the V1 dermatome, often with Hutchinson’s sign (nasal tip involvement), which is predictive of ocular complications. Chronic sequelae such as neurotrophic keratitis and corneal scarring can lead to significant visual morbidity.
5. Neuro-Ophthalmic Disorders: HIV affects the central and peripheral nervous systems, often resulting in neuro-ophthalmic complications due to opportunistic infections, direct viral invasion, or mass lesions. Key conditions are optic neuropathy, cranial nerve palsies, and cortical visual impairment. These disorders may be irreversible, especially when diagnosis and treatment are delayed. Neuroimaging, lumbar puncture, and prompt antimicrobial therapy are crucial for management.
6. HIV-Related Ocular Tumors: PLWH are at increased risk of certain malignancies due to chronic immune dysregulation and coinfections with oncogenic viruses like HHV-8 and EBV. Common tumors are Kaposi Sarcoma, Primary Intraocular Lymphoma (PIOL), Orbital and CNS Lymphomas. Treatment typically involves chemotherapy, radiotherapy, and systemic HAART. Ocular tumors in HIV can result in significant, sometimes irreversible visual loss, and early detection remains a challenge due to their masquerade presentation.

The spectrum of HIV-related ocular disease is vast, ranging from mild, asymptomatic retinopathy to rapidly blinding infections and malignancies. In the era of HAART, opportunistic infections remain a major cause of low vision, particularly in settings with delayed HIV diagnosis or poor treatment adherence. Neuro-ophthalmic and tumor-related complications may also result in profound and permanent visual disability. Timely diagnosis, multidisciplinary management, and integration of ocular care into HIV programs are essential to reducing the burden of low vision in this population.

### Mechanisms of Vision Loss in HIV

Vision loss in people living with HIV (PLWH) arises through several interrelated mechanisms, including direct viral effects, immune-mediated damage, opportunistic infections, and treatment-associated toxicities. These processes may affect various levels of the visual system—from the retina and optic nerve to the cortical visual centers. Understanding the underlying pathophysiology is critical for timely intervention and prevention of irreversible low vision or blindness [2-6].

1. Retinal Destruction

Opportunistic infections and microvascular disease most commonly cause retinal damage in HIV-positive individuals. Among these, cytomegalovirus (CMV) retinitis stands out as the leading cause of retinal necrosis and subsequent blindness in AIDS patients.

Mechanism:

- CMV invades retinal cells, causing full-thickness necrosis of retinal tissue.
- The inflammatory response exacerbates retinal edema, hemorrhage, and ischemia.
- Late-stage complications include retinal detachment, scarring, and neovascularization.

In untreated CMV retinitis, vision can deteriorate rapidly within weeks, especially when the macula or optic disc is involved. Even after antiviral therapy, scarring and fibrosis can leave permanent central vision loss or peripheral field defects.

Non-infectious HIV retinopathy also contributes to retinal damage through:

- Capillary dropout and ischemia;
- Microaneurysms;
- Cotton wool spots from axoplasmic stasis;
- Blood-retinal barrier breakdown.

While often subclinical, advanced HIV retinopathy may evolve into macular ischemia and thinning, leading to reduced visual acuity over time.

2. Optic Neuropathy

The optic nerve is highly vulnerable in PLWH, and damage may occur from direct viral involvement, infections, malignancy, or toxic insults. Optic neuropathy manifests as reduced visual acuity, afferent pupillary defect, color vision loss, and visual field defects.

Etiologies include:

- HIV-associated optic neuritis (rare but reported, particularly in the absence of infection);
- Infectious causes:
  - MV, syphilis, tuberculosis, cryptococcus, and herpes viruses.
- Compressive optic neuropathy due to:
  - Orbital or CNS lymphoma;
  - Opportunistic mass lesions (e.g., tuberculomas).
  Pathogenesis:
- Demyelination and axonal injury due to direct infection or secondary immune-mediated inflammation.
- Vascular compromise leading to anterior ischemic optic neuropathy (AION).
- Chronic elevation of intracranial pressure in meningitis can also cause papilledema and secondary optic atrophy.

Optic nerve damage is often irreversible, especially when diagnosis is delayed or when concurrent retinal disease obscures early detection.

3. Cortical Visual Pathway Involvement

In some PLWH, vision loss results not from ocular pathology but from lesions involving the visual pathways in the brain. This cortical visual impairment (CVI) may present as unexplained bilateral visual loss with normal fundus examination.

Common causes:

- Progressive multifocal leukoencephalopathy (PML) due to JC virus reactivation, causing demyelination in occipital or parietal lobes.
- HIV encephalopathy (HIV-associated neurocognitive disorder) affecting the visual association cortex.
- CNS toxoplasmosis, tuberculomas, or lymphomas compressing the optic radiations or occipital cortex.

Symptoms include:

- Homonymous hemianopia;
- Visual agnosia;
- Cortical blindness (loss of vision with preserved pupillary light reflexes).

Neuroimaging (MRI) is essential for diagnosis. Unfortunately, cortical damage is often permanent, and rehabilitation is challenging due to the injury’s central location.

4. Drug-Related Ocular Toxicity

With the advent of highly active antiretroviral therapy (HAART), the spectrum of ocular complications has shifted, with drug-induced toxicity emerging as an important but underrecognized cause of vision loss.

Key offending agents:

- Didanosine (ddI):
  - Associated with retinal pigment epitheliopathy and peripheral chorioretinal atrophy, possibly due to mitochondrial toxicity.
  - Symptoms: night blindness, peripheral visual field loss.
- Rifabutin (used for Mycobacterium avium complex):
  - Can cause uveitis, hypopyon, or retinal vasculitis.
- Cidofovir:
  - Used in CMV retinitis but may induce uveitis and hypotony, necessitating corticosteroid cover.
- Ethambutol (used in TB coinfection):
  - Causes toxic optic neuropathy, with bilateral central vision loss, dyschromatopsia, and central scotomas.
  - Risk increases with higher doses and renal impairment.
- Chloroquine/Hydroxychloroquine:
  - Occasionally used for autoimmune conditions in HIV; risk of bull’s eye maculopathy with prolonged use.

Mechanisms:

- Mitochondrial dysfunction in retinal ganglion cells or RPE;
- Direct neurotoxicity to optic nerve fibers;
- Hypersensitivity or idiosyncratic immune-mediated reactions.

Monitoring for ocular toxicity, especially in patients on long-term multidrug regimens or with pre-existing eye disease, is essential. Periodic visual field testing, OCT imaging, and fundus evaluation should be part of standard follow-up.

Vision loss in HIV is multifactorial, stemming from infectious destruction of the retina, optic nerve compromise, cortical damage, and iatrogenic toxicity. While some causes are preventable or treatable when detected early, others may lead to irreversible blindness. A multidisciplinary approach that includes ophthalmologists, neurologists, and infectious disease specialists is critical to minimizing visual disability in HIV-infected individuals.

### Diagnosis of Ocular Complications in HIV

Timely diagnosis of ocular disease in people living with HIV (PLWH) is crucial to prevent irreversible visual loss. Due to the wide range of possible etiologies—ranging from opportunistic infections and malignancies to drug-induced toxicities—a structured, multimodal diagnostic approach is essential. The diagnostic process combines clinical examination, imaging, laboratory testing, and increasingly, teleophthalmology-based support [3-7].

1. Clinical Tools

A detailed clinical evaluation remains the cornerstone of diagnosing ocular manifestations of HIV. This includes:

- History Taking:
  - Onset and progression of symptoms (e.g., blurring, floaters, photophobia, pain, diplopia).
  - History of opportunistic infections (e.g., CMV, TB, toxoplasmosis).
  - Medication history (e.g., use of ethambutol, didanosine).
  - Immune status, particularly CD4 count, ART adherence, and history of immune reconstitution inflammatory syndrome (IRIS).
- Visual Acuity Testing:
  - Standardized Snellen or LogMAR charts to assess baseline vision.
- Slit Lamp Biomicroscopy:
  - To examine anterior segment inflammation (e.g., keratic precipitates, iritis).
- Dilated Fundus Examination:
  - Direct and indirect ophthalmoscopy for retinal lesions (e.g., hemorrhages, cotton-wool spots, retinal necrosis).
- Intraocular Pressure (IOP):
  - Measured to detect uveitis-related hypotony or steroid-induced glaucoma.
- Pupillary Reflex and Color Vision Tests:
  - Evaluate optic nerve function (e.g., afferent pupillary defect, red desaturation).

In resource-limited settings, a basic clinical toolkit can be life-saving if frontline providers are trained to recognize hallmark features of HIV-related eye disease.

2. Imaging Techniques

Imaging is indispensable for evaluating the retina, optic nerve, and posterior segment in suspected ocular HIV cases:

- Fundus Photography:
  - Useful for documenting baseline and monitoring progression (especially for CMV retinitis and HIV retinopathy).
- Optical Coherence Tomography (OCT):
  - Non-invasive imaging of retinal architecture.
  - Detects macular edema, epiretinal membranes, and retinal thinning.
  - Useful in identifying drug-induced retinal toxicity (e.g., didanosine-related atrophy).
- Fluorescein Angiography (FA):
  - Evaluates retinal vasculature for ischemia, leakage, and neovascularization.
  - Helps differentiate CMV retinitis from HIV retinopathy or toxoplasmosis.
- Ultrasound B-scan:
  - Important in cases with media opacities (e.g., dense vitritis or cataract).
  - Can detect retinal detachment or intraocular masses.
- MRI of the Brain and Orbits:
  - Indicated when neuro-ophthalmic symptoms are present.
  - Useful in diagnosing cortical blindness, optic neuritis, CNS toxoplasmosis, or lymphoma.

Advanced imaging is particularly critical in immunosuppressed patients, where fundus findings may be subtle and fast progression can occur.

3. Laboratory Analysis

Laboratory investigations are essential for confirming infectious etiologies, guiding treatment, and assessing systemic disease control:

- CD4 Count:
  - A low CD4 count (< 50 cells/μL) increases risk for CMV retinitis and other opportunistic infections.
- HIV Viral Load:
  - Useful in correlating immune status with ocular disease risk.
- PCR Testing of Aqueous/Vitreous Fluid:
  - Detects DNA of organisms such as CMV, HSV, VZV, or Toxoplasma gondii.
  - Gold standard in diagnosing infectious posterior uveitis or retinitis.
- Serological Tests:
  - Toxoplasma IgG/IgM, syphilis serology (VDRL, RPR, FTA-ABS), TB screening (Quantiferon Gold or Mantoux), and cryptococcal antigen.
- CSF Analysis:
  - Required if optic neuritis or meningitis is suspected.
- Cytology and Flow Cytometry:
  - In suspected intraocular lymphoma, an anterior chamber or vitreous tap may be analyzed.

Laboratory workup should be tailored to ocular findings, immune status, and endemic infections.

4. Role of Teleophthalmology

Teleophthalmology is increasingly recognized as a significant change in managing ocular HIV, particularly in remote and underserved regions:

- Digital Retinal Imaging with Remote Review:
  - Trained technicians can acquire fundus photographs and transmit them to specialists for interpretation.
  - Useful for CMV retinitis screening in HIV clinics.
- Smartphone-Based Fundus Photography:
  - Low-cost alternative using mobile phones and adaptors, useful for point-of-care triage.
- Virtual Consultations:
  - Patients in peripheral centers can consult ophthalmologists via video conferencing.
- AI-Based Image Analysis:
  - Emerging algorithms can detect retinal lesions from photographs, aiding in early diagnosis and referral.

Benefits:

- Reduces diagnostic delays.
- Bridges the specialist gap in rural or resource-poor settings.
- Allows longitudinal monitoring of chronic disease (e.g., HIV retinopathy or optic atrophy).

Limitations:

- Dependence on image quality and trained personnel.
- May miss subtle anterior segment or neuro-ophthalmic signs.

Despite limitations, teleophthalmology offers a scalable and sustainable approach to integrated HIV and eye care delivery, especially in low-income countries.

An accurate and timely diagnosis of HIV-related ocular disease requires an integrative approach—merging bedside clinical acumen with modern imaging, laboratory diagnostics, and telehealth innovations. Early detection is key to preserving vision and quality of life in PLWH, and embedding ophthalmologic evaluation into routine HIV care pathways can significantly improve outcomes.

### Management Strategies for HIV-Related Low Vision

The management of low vision in people living with HIV (PLWH) demands a multidisciplinary and individualized approach, addressing not only the underlying ocular pathology but also the functional consequences of vision loss. As HIV has transitioned from a terminal illness to a chronic, manageable condition, the focus has shifted toward improving quality of life, including visual rehabilitation and integration of eye care into long-term HIV management [13-16].

1. Medical Management of Ocular Complications

- Antiretroviral Therapy (ART)
  The cornerstone of preventing and managing HIV-related eye disease is effective systemic control of the virus:
  - Early initiation and adherence to ART reduces the incidence and recurrence of opportunistic infections such as CMV retinitis, toxoplasmosis, and others.
  - ART also lowers the risk of HIV-associated neuroretinal disorders and IRIS.
  - Some ocular manifestations, including HIV retinopathy and optic neuropathy, may regress or stabilize once ART is optimized.
- Specific Treatment for Opportunistic Infections
  - CMV Retinitis: Treated with systemic (oral valganciclovir, IV ganciclovir/foscarnet) and intravitreal antiviral injections. Early treatment can preserve vision; however, delayed diagnosis often results in irreversible retinal destruction.
  - Ocular Toxoplasmosis: Treated with a combination of pyrimethamine, sulfadiazine, and folinic acid or trimethoprim-sulfamethoxazole, often with adjunctive steroids.
  - Herpes Zoster Ophthalmicus: Requires systemic acyclovir or valacyclovir; topical steroids and cycloplegics may help with anterior uveitis.
  - Tuberculosis and Syphilis: Require prolonged systemic antimicrobial therapy guided by local resistance patterns and CNS involvement.
  - Fungal Infections (e.g., Cryptococcus): Managed with systemic antifungals (amphotericin B, fluconazole) and control of intracranial pressure if the optic nerve is affected.
- Immune Reconstitution Inflammatory Syndrome (IRIS)

Ocular IRIS can present as worsening uveitis or vitritis after ART initiation. Management includes:

- Observation in mild cases;
- Topical or systemic corticosteroids in severe inflammation;
- Continuation of ART unless vision is acutely threatened.

2. Surgical and Interventional Options

- Retinal Detachment Repair: Common in advanced CMV retinitis; managed surgically via vitrectomy, scleral buckle, or silicone oil tamponade.
- Cataract Surgery: Frequently indicated due to steroid use or HIV-related uveitis. Careful pre-op evaluation for inflammation and infection is essential.
- Glaucoma Surgery: In steroid-induced or inflammatory glaucoma, trabeculectomy or tube shunt may be required.
- Ocular Tumor Management: For conditions like Kaposi’s sarcoma or lymphoma, treatment may involve systemic chemotherapy, radiotherapy, or localized excision, in consultation with oncology.

3. Low Vision Rehabilitation

When vision loss becomes permanent, rehabilitation aims to maximize remaining vision and functionality:

- Visual Aids and Assistive Devices
  - Magnifiers (hand-held or stand-mounted);
  - Telescopic glasses for distance viewing;
  - High-contrast reading materials, talking watches, screen readers, and Braille displays;
  - Smartphone apps and AI-powered visual assistants (e.g., Seeing AI, Be My Eyes).
- Orientation and Mobility Training
  - Teaching navigation skills using canes, auditory cues, or trained guide assistance.
  - Helpful for patients with central vision loss (e.g., macular involvement in CMV).
- Environmental Modifications
  - Enhanced lighting, contrast-enhanced settings at home, tactile labels on objects.
  - Voice-activated appliances and accessible digital platforms.
- Psychosocial Support
  - Counseling, peer support groups, and occupational therapy are vital to address the psychological toll of dual stigma from HIV and vision impairment.
  - Education of caregivers and community-based support improves adaptation and compliance.

4. Preventive and Public Health Measures

- Routine Ophthalmic Screening
  - Annual eye exams are recommended for PLWH, particularly those with CD4 < 200 cells/μL.
  - Fundus photography and OCT screening for asymptomatic retinopathy can detect early disease.
- Integration with HIV Care Services
  - Eye screening should be part of comprehensive HIV care, especially in ART clinics.
  - Training primary care and HIV clinicians to identify red flags for ocular referral.
- Patient Education
  - Promoting awareness about early symptoms like floaters, vision blurring, and photophobia.
  - Emphasizing ART adherence and the importance of reporting eye symptoms.
- Vaccination and Prophylaxis
  - Varicella vaccination in non-immune adults may reduce the risk of Herpes Zoster.
  - Primary prophylaxis with TMP-SMX reduces toxoplasmosis risk.
  - Ganciclovir prophylaxis in high-risk patients (CD4 < 50) may prevent CMV retinitis.

5. Innovations in Care Delivery

- Teleophthalmology-Based Interventions
  - Remote monitoring of retinitis or optic neuropathy in rural areas using smartphone-based fundoscopy or portable OCT.
  - Virtual follow-ups reduce travel burden and improve access.
- Artificial Intelligence and Deep Learning
  - AI algorithms trained to detect CMV retinitis or retinal hemorrhages from fundus photos can aid frontline workers in early triage.
- Mobile Clinics and Community Eye Camps
  - Especially useful in high HIV-burden, low-resource regions.
  - Integration with ART distribution and HIV screening services improves coverage.

6. Multidisciplinary Collaboration

Managing HIV-related low vision requires cooperation among:

- Ophthalmologists;
- Infectious disease specialists;
- Neurologists;
- Low vision therapists;
- Community health workers;
- Mental health professionals.

Such collaboration ensures early detection, appropriate intervention, and long-term functional support. Effective management of HIV-related low vision extends beyond ocular treatment. It encompasses systemic HIV control, timely management of opportunistic infections, rehabilitative support, and innovative service delivery. With the growing aging HIV-positive population, a proactive approach to eye health is not just a clinical necessity but a human rights imperative.

### Low Vision Rehabilitation in HIV

As HIV transitions into a chronic condition with improved survival due to widespread use of antiretroviral therapy (ART), the burden of irreversible visual impairment among people living with HIV (PLWH) has become increasingly apparent. In such cases, low vision rehabilitation offers an opportunity to enhance quality of life, restore independence, and improve psychosocial well-being. However, rehabilitation for HIV-related vision loss requires tailored strategies that address both the functional deficits and the broader context of HIV care [7-9,11-13].

1. Strategies and Devices for Visual Rehabilitation

Low vision rehabilitation is a multidisciplinary effort aimed at optimizing residual vision using a combination of assistive technologies, environmental adaptations, training, and psychosocial support. In PLWH with irreversible ocular damage—due to CMV retinitis, optic atrophy, or cortical visual impairment—rehabilitation can restore functional independence and reduce disability.

- Optical and Non-optical Devices
  - Hand-held and stand magnifiers: Useful for reading and close tasks.
  - High-powered reading glasses: Often prescribed for near vision activities.
  - Telescopic spectacles or monoculars: Enable distance vision tasks such as viewing signage or presentations.
  - Video magnifiers (CCTV devices): Offer electronic zooming, contrast enhancement, and color inversion.
  - Smartphone apps and screen readers: Applications such as VoiceOver, TalkBack, Seeing AI, and Be My Eyes assist with text recognition, navigation, and real-time interpretation.
  - Braille devices and tactile aids: Particularly helpful for individuals with profound visual loss or dual sensory impairment.
  - Contrast-enhanced and high-illumination tools: Task lighting and high-contrast materials (e.g., large-font labels) aid visibility and reduce strain.
- Orientation and Mobility Training
  Patients with field defects or severe visual acuity loss benefit from:
  - White cane training for independent navigation.
  - Environmental orientation skills to maneuver safely in homes, public spaces, or workplaces.
  - Auditory cues and echolocation techniques to replace lost visual input.
- Environmental Modifications
  Modifications in home and work environments help optimize safety and functionality:
  - Use of contrasting colors (e.g., dark plates on light tables);
  - Non-slip mats and handrails;
  - Voice-controlled appliances;
  - Labeling objects with large print or Braille;
  - Voice assistants like Alexa or Google Assistant for calendar, reminders, and reading tasks.

2. Integration with HIV Care

Effective visual rehabilitation must be integrated into the continuum of HIV care, particularly in resource-limited settings. Many PLWH attend HIV clinics regularly, offering a unique opportunity to screen, refer, and provide vision services within the same care framework.

- Screening and Referral
  - Routine visual acuity checks and basic fundoscopy should be included in ART clinic protocols, especially in patients with CD4 < 200 cells/μL or a history of ocular complaints.
  - Training HIV clinicians and nurses to identify “red flag” symptoms such as floaters, scotomas, or photophobia can facilitate timely referrals.
  - Integration of vision screening forms into electronic medical records ensures systematic evaluation.
- Co-location of Services
  - Co-locating low vision services within HIV care facilities (e.g., tertiary hospitals or district ART centers) minimizes travel and stigma-related barriers.
  - Eye camps organized alongside HIV outreach services (e.g., mobile clinics) can screen and dispense optical aids in underserved areas.
- Inclusion in National HIV Programs
  - National HIV control programs should include visual rehabilitation in their disability support schemes.
  - Funding support for low vision devices, similar to ART and nutritional supplements, is essential.
  - Inclusion of low vision rehabilitation in training curricula for HIV care providers ensures a more integrated approach to patient management.

### Psychosocial Consequences and Stigma in HIV-Related Low Vision

The intersection of HIV and low vision presents a unique and often debilitating dual burden—one rooted not only in clinical impairment but also in deeply entrenched social stigma. While each condition individually can affect a person’s mental health and social integration, their co-occurrence exacerbates psychological distress, impedes care-seeking behavior, and diminishes overall quality of life. A holistic understanding of these psychosocial dimensions is critical to delivering compassionate and comprehensive care for people living with HIV (PLWH) who experience visual disability [14-16].

1. Dual Burden of Disease

Living with HIV carries significant emotional and societal challenges. When combined with visual impairment—often irreversible—the challenges become compounded. The dual burden is experienced on several levels:

- Physical dependence: Loss of vision can compromise an individual’s ability to perform daily activities such as reading medication labels, navigating transportation, or managing financial tasks. These limitations become especially problematic for PLWH, who must adhere to complex ART regimens and engage frequently with health services.
- Social exclusion: Visual impairment can cause withdrawal from social interactions due to fear of ridicule, increased dependency, and restricted mobility. For PLWH, who may already feel isolated due to their HIV status, this leads to amplified marginalization.
- Economic vulnerability: HIV and vision loss both disproportionately affect people in their most productive years. The combination often leads to loss of employment, inability to secure stable income, and increased caregiver dependence, particularly in resource-limited settings where vocational opportunities for people with disabilities are minimal.

2. Depression, Anxiety, and Quality of Life

Numerous studies have documented higher rates of depression and anxiety in people with either HIV or low vision. When both conditions coexist, mental health outcomes are significantly worse:

- A study in Kenya reported that over 50% of HIV-positive individuals with visual impairment exhibited signs of moderate to severe depression.
- In South Africa, women with both HIV and low vision were found to have lower quality of life scores, particularly in domains related to self-care, social functioning, and emotional well-being.
- Cognitive impairment associated with HIV can further complicate adaptation to vision loss, making rehabilitation more challenging and psychological adjustment more complex.

Key psychological consequences include:

- Hopelessness and helplessness due to loss of independence;
- Fear of abandonment by family or partners;
- Internalized stigma, where individuals believe they are “less than” or “invisible” to society;
- Post-traumatic stress, especially in those who acquired visual impairment due to sudden events like CMV retinitis or stroke.

3. Counseling and Peer-Support Networks

Addressing these psychosocial consequences requires integrated, community-centered approaches:

- Counseling Services
  - Individual psychotherapy can address depression, trauma, and adjustment to vision loss. Cognitive behavioral therapy (CBT) has been shown to improve coping in PLWH.
  - Pre- and post-diagnosis counseling should include discussions about the risk of ocular complications and ways to maintain independence.
  - Grief counseling is vital for those who experience sudden or progressive vision loss.
- Peer Support and Rehabilitation Networks
  - Peer-led support groups allow individuals to share experiences, exchange practical tips (e.g., for navigating mobility or managing ART with vision loss), and reduce feelings of isolation.
  - Mentorship models, where individuals with stable vision impairment counsel newly diagnosed patients, have been shown to improve emotional resilience significantly.
  - Community-based organizations (e.g., HIV NGOs, vision advocacy groups) can play a key role in forming these networks.
- Psychosocial Rehabilitation Integration
  - Low vision rehabilitation should include a psychosocial module that evaluates mental health, addresses coping strategies, and encourages social reintegration.
  - Social workers and rehabilitation psychologists must be part of interdisciplinary care teams for PLWH with low vision.

#### Tackling Stigma: A Multilevel Challenge

Stigma exists at multiple levels:

- Societal stigma: Discrimination from the community, employers, and even healthcare workers.
- Family stigma: Rejection or overprotection, particularly of women and children.
- Institutional stigma: Lack of accessibility in public services, educational systems, or health programs.
- Internalized stigma: Guilt, shame, or self-blame.

To overcome stigma:

- Awareness campaigns must emphasize that visual impairment and HIV are manageable conditions and not a reflection of moral failure or weakness.
- Education of healthcare providers is essential to eliminate bias and ensure respectful, inclusive care.
- Legal frameworks must enforce protections for disabled people and those living with HIV, including workplace accommodations and equal rights.

The psychosocial impact of HIV-related low vision extends far beyond clinical symptoms. It involves complex layers of stigma, emotional distress, and social dislocation. Without deliberate efforts to address these facets, even the best medical and rehabilitative interventions will fall short. By fostering peer networks, integrating mental health services, and promoting inclusive, nonjudgmental care, we can support PLWH not only to survive—but to thrive despite visual disability.

### Global Health Perspectives on HIV And Low Vision

The burden of HIV-related visual impairment is not just a medical issue but a global public health challenge. Addressing it effectively requires coordinated strategies that incorporate integrated care models, community outreach, and international health policy support. Although many advances have been made in HIV management, the integration of eye health within HIV care frameworks remains an underutilized opportunity. Several global health organizations, including the World Health Organization (WHO) and UNAIDS, have acknowledged this need and proposed policies and programs that are beginning to shape comprehensive care delivery in both high- and low-income settings [17-20].

1. Models of Integrated Eye and HIV Care

Efficient models of integrated care for HIV and eye health are still evolving but have demonstrated promising results, especially in resource-limited regions.

- Clinic-Based Integration
  In high-volume HIV clinics, integrating routine ophthalmic screening into HIV care has proven cost-effective and scalable. For example:
  - In Botswana, HIV clinics embedded visual acuity screening and dilated fundoscopy for patients with CD4 counts below 200 cells/μL, resulting in earlier detection of CMV retinitis and HIV retinopathy.
  - In Thailand, task-shifting models allowed trained nurses to conduct basic ocular screenings for early referral, increasing access to eye care in remote HIV-positive populations.
- One-Stop Multispecialty Clinics
  Urban centers in South Africa and Brazil have piloted multidisciplinary clinics in which ophthalmologists, infectious disease specialists, neurologists, and psychologists collaborate. These clinics facilitate:
  - Timely diagnosis and management of ocular complications.
  - Streamlined ART monitoring and visual rehabilitation.
  - Counseling for both HIV-related stigma and vision loss.
- Digital Integration

With the rise of electronic health records (EHRs), many programs now include ocular health data in comprehensive patient records within HIV care systems. These foster coordinated care, especially in tertiary centers.

2. Community-Based Screening Programs

Given the high prevalence of HIV and the growing risk of visual disability in many low- and middle-income countries (LMICs), community-based outreach is essential for early detection and intervention.

- Mobile Eye Units
  Countries such as Kenya, India, and Nigeria have deployed mobile eye care clinics that visit HIV treatment centers, prisons, and rural communities. These units provide:
  - Visual acuity testing and refraction.
  - Fundus imaging and slit-lamp exams.
  - Low vision aid distribution and referrals.
- Vision Screening by Community Health Workers (CHWs)
  Training CHWs to recognize early symptoms of ocular involvement in HIV has shown encouraging results. CHWs can:
  - Identify red flags (e.g., floaters, blurred vision, ocular pain).
  - Refer high-risk individuals (e.g., with low CD4 count or ART failure).
  - Support medication adherence and follow-up appointments.
- Integrated HIV and Eye Camps
  In rural sub-Saharan Africa and South Asia, eye camps have been successfully merged with HIV testing and counseling services, destigmatizing both services and improving uptake. These camps often include:
  - Cataract surgeries for HIV-positive individuals.
  - On-site CD4 and viral load testing.
  - Educational workshops on HIV and eye health.

3. WHO and UNAIDS Policies and Frameworks

Both WHO and UNAIDS have outlined strategic frameworks that support integration of vision care into HIV services, though implementation remains uneven globally.

- WHO: Integrated People-Centered Eye Care (IPEC)
  - The IPEC framework (2020) emphasizes the need to embed eye care into broader health systems, particularly at the primary level.
  - It recommends screening high-risk populations, including those with chronic diseases like HIV, diabetes, and TB.
  - WHO supports the development of national eye health plans that align with existing HIV infrastructure.
- WHO: Package of Essential Noncommunicable (PEN) Interventions
  - This package includes basic eye assessments as part of chronic disease care.
  - It encourages HIV clinics to offer vision screening during ART follow-ups.
- UNAIDS Global AIDS Strategy (2021-2026)
  - Although not explicitly eye-focused, the strategy calls for integrated service delivery and reaching underserved populations with comprehensive, stigma-free care.
  - It encourages cross-sectoral collaboration, including with rehabilitation and disability sectors, making room for eye care partnerships.
- UN Convention on the Rights of Persons with Disabilities (CRPD)
  - This treaty, ratified by over 180 countries, mandates equal access to healthcare and rehabilitation services for people with disabilities, including visual impairment.
  - It applies fully to PLWH with low vision and supports inclusive policy-making.

### Challenges in Policy Implementation

Despite supportive global frameworks, barriers persist:

- Fragmented funding often separates HIV and eye health programs.
- Lack of awareness about ocular manifestations of HIV among policymakers and program directors.
- Shortage of trained ophthalmic personnel, particularly in rural HIV-prevalent regions.
- Stigma and fear continue to deter people from accessing services.

### Global Call to Action

To address these gaps, global health stakeholders must prioritize:

- Funding integrated service models that combine HIV care with vision screening and rehabilitation.
- Training front-line workers in both domains.
- Expanding community-based outreach to include people living with disabilities.
- Monitoring and evaluation frameworks that measure ocular health outcomes in HIV-positive populations.

Global health perspectives underscore the urgent need to bridge HIV and eye care, especially as the HIV-positive population ages and faces chronic comorbidities. By embracing integrated, community-based, and rights-based approaches, the global community can better ensure that no person living with HIV is left in the dark—literally or figuratively—due to preventable or treatable vision loss.

### Future Directions and Research Gaps

The interplay between HIV and low vision represents a complex and evolving frontier in global health. While antiretroviral therapy (ART) has dramatically improved the life expectancy of people living with HIV (PLWH), the long-term ocular sequelae of HIV infection, opportunistic infections, and drug toxicities remain underexplored. The current body of literature reflects significant knowledge gaps, especially regarding epidemiology, prevention, and management of visual disability in this population. Future research and policy must aim to close these gaps through interdisciplinary collaboration and inclusive healthcare delivery models.

1. Need for Large-Scale Epidemiological Studies

Although various studies have documented ocular manifestations in HIV, there is a lack of large-scale, population-based data quantifying the prevalence and incidence of low vision in PLWH—especially in pediatric and geriatric subgroups.

Future focus areas include:

- Prevalence and risk factors of irreversible visual impairment among HIV-infected individuals on long-term ART.
- Comparative data between high-income and low-resource settings.
- Ethnic, gender, and geographic disparities in visual outcomes.

2. Understanding Pathophysiological Mechanisms

There is a pressing need to elucidate the molecular and immunological pathways through which HIV and ART drugs contribute to ocular degeneration. Understanding the pathogenesis of HIV-associated retinopathy, optic neuropathy, and cortical visual impairment can guide novel interventions.

Key areas include:

- Role of chronic immune activation and microvascular damage in the retina.
- Long-term effects of ART on the optic nerve and retinal ganglion cells.
- Mechanisms of neurotoxicity from HIV or associated co-infections.

3. Technological Innovations for Diagnosis and Screening

Advancements in digital imaging, artificial intelligence (AI), and teleophthalmology can revolutionize early detection of vision-threatening conditions in PLWH, especially in underserved regions.

Promising research directions include:

- Development of AI-based tools for identifying HIV-related retinal changes.
- Use of portable fundus cameras integrated into HIV care settings.
- Mobile apps for self-assessment of visual symptoms.

Research should focus on validating these tools and integrating them into existing HIV service frameworks.

4. Vision Rehabilitation: Evidence-Based Interventions

While low vision rehabilitation is well established in general ophthalmology, tailored rehabilitation strategies for HIV-related vision loss are underdeveloped.

Unanswered questions:

- What are the most effective visual aids for PLWH with concurrent systemic comorbidities?
- Can low vision devices be integrated into ART delivery programs?
- What is the impact of multidisciplinary rehabilitation on long-term quality of life?

Prospective studies are needed to evaluate the cost-effectiveness, acceptability, and sustainability of these interventions in resource-poor settings.

5. Pediatric and Geriatric Visual Outcomes in HIV

Children infected with HIV may suffer from developmental delays and cortical visual impairment, while older adults with HIV may face compounded risks from age-related eye diseases.

Research gaps include:

- Longitudinal visual outcomes of perinatally infected children.
- Optimal screening intervals and tools for elderly PLWH.
- School-based and home-based vision support for pediatric HIV populations.

6. Policy and Programmatic Research

The lack of integrated service delivery remains one of the largest barriers to optimal care. There is a need for implementation research that informs:

- How best to incorporate eye care into national HIV programs?
- Which funding models support integrated care without overburdening providers?
- How to train primary healthcare workers in dual disease recognition.

Pilot programs in Africa and Southeast Asia can serve as models for scalable integration.

7. Psychosocial Impact and Stigma Reduction

Dual stigma—from both HIV and visual impairment—negatively affects mental health and healthcare-seeking behavior. More research is needed into:

- The psychosocial burden of combined diagnoses.
- Effective models of peer support and psychosocial counseling.
- Community education strategies to reduce discrimination and isolation.

Quantitative and qualitative studies can help design culturally sensitive interventions that resonate with affected populations.

8. Collaboration and Data Sharing

HIV and low vision span multiple disciplines—infectious disease, neurology, ophthalmology, rehabilitation, mental health, and public health. A multisectoral approach is essential to generating robust data and delivering holistic care.

Future directions:

- Establishing global registries for ocular complications in PLWH.
- Creating interdisciplinary research networks and consortia.
- Encouraging data sharing through open-access platforms and WHO/UNAIDS collaborations.

The confluence of HIV and low vision represents an emerging global health priority that demands increased attention, investment, and innovation. As HIV transitions from a fatal disease to a chronic condition, the focus must expand from mere survival to quality of life and functional well-being. Addressing low vision in HIV-positive populations is not only a clinical imperative but also a human rights issue—encompassing access to care, dignity, and inclusion.

The path forward lies in integrated models of care, community-based screening, research-driven rehabilitation, and stigma-free service delivery. With robust political will, global collaboration, and sustained advocacy, it is possible to ensure that no individual living with HIV or AIDS experiences preventable vision impairment or is denied access to quality eye care and treatment.

## Conclusion

HIV can lead to significant visual impairment through direct viral effects, opportunistic infections, and treatment-related complications. As life expectancy increases with ART, vision-related issues in people living with HIV (PLWH) are becoming more common and impactful. Despite this, vision care is often overlooked in HIV programs. Integrated eye care, early screening, and low vision rehabilitation are essential. Addressing psychosocial challenges like stigma and depression is also critical. Ensuring visual health in PLWH is a key step toward comprehensive, equitable, and dignified HIV care.
